# Golden hour management of infants with congenital diaphragmatic hernia: 15 year experience at a high-volume center

**DOI:** 10.1038/s41372-025-02226-z

**Published:** 2025-02-21

**Authors:** K. Taylor Wild, Holly L. Hedrick, Natalie E. Rintoul, Anne M. Ades, Juliana S. Gebb, Leny Mathew, Tom Reynolds, Anna Bostwick, Elizabeth Eppley, Sabrina Flohr, N. Scott Adzick, Elizabeth E. Foglia

**Affiliations:** 1 Division of Neonatology, Children’s Hospital of Philadelphia, Perelman School of Medicine at University of Pennsylvania, Philadelphia, PA USA; 2 Richard D. Wood Center for Fetal Diagnosis and Treatment, Children’s Hospital of Philadelphia, Philadelphia, PA USA; 3 Division of Pediatric General, Thoracic, and Fetal Surgery, Children’s Hospital of Philadelphia, Perelman School of Medicine at University of Pennsylvania, Philadelphia, PA USA

**Keywords:** Paediatrics, Prognosis

## Abstract

**Objective:**

To review the evolution of golden hour management and outcomes for infants with congenital diaphragmatic hernia (CDH).

**Study design:**

Retrospective single center cohort study of infants with CDH born 2008–2023 at a quaternary children’s hospital. Infants were grouped into 3 epochs: 2008–2013, 2014–2018, and 2019–2023. Outcome measures included extracorporeal membrane oxygenation therapy and survival.

**Result:**

There were 454 infants, including 106 (2008–2013), 156 (2014–2018), and 192 (2019–2023). Despite increased disease severity, survival improved over time, from 71% (2008–2013) to 82% (2014–2018) and 83% (2019–2023), *p* = 0.02 for trend, with no difference in ECMO utilization.

**Conclusion:**

Management of infants with CDH continues to evolve with ongoing experience at our high-volume center. Despite increasing severity of illness, survival outcomes have improved over time. In the absence of clinical trial data, observational data should be evaluated rigorously to inform care in a data-driven manner.

## Introduction

Congenital diaphragmatic hernia (CDH) occurs when the fetal abdominal viscera herniate into the thoracic cavity through a diaphragmatic defect. Pulmonary hypoplasia and pulmonary hypertension may occur as a result of this defect and may complicate the typical postnatal cardiorespiratory transition. Effective delivery room (DR) resuscitation of infants with CDH is complex and requires critical interventions to occur simultaneously or in quick succession [1]. Rapid intubation and early gentle ventilation optimize gas exchange and are associated with improved survival [2–5]. Annibale et al. introduced the concept of adapting the Golden Hour of Trauma to a golden hour of DR management in preterm infants from birth through initial stabilization [6]. The goal of the golden hour is to rapidly stabilize an infant with critical interventions and systematic decision making. While initially described for the DR management of preterm infants, the concept of a golden hour of stabilization can be extrapolated to infants with complex congenital anomalies like CDH.

Existing CDH management guidelines are based predominantly on expert opinion, particularly regarding DR management [7–11]. Large trials are lacking in this population, and many changes in DR management strategies have occurred based solely on observational data and expert opinion.

The Garbose Family Special Delivery Unit (SDU) at the Children’s Hospital of Philadelphia is the world’s first birth facility located in a children’s hospital, allowing infants with congenital anomalies to be born with immediate access to quaternary level care [12, 13]. Each year, there are approximately 500 deliveries in the SDU, including about 50 infants with CDH [14].

The objective of this study was to leverage this large single center experience delivering and managing infants with CDH to review the evolution of golden hour care and outcomes.

## Methods

### Design

This was a retrospective single center cohort study of infants with CDH born 2008–2023. We have structured our CDH DR program using the framework of a Learning Health System [15] to inform and improve care. In this model, observations of clinical performance and outcomes prompted changes to clinical management, which were then assessed through ongoing review of patient data. Temporal changes in golden hour management included initiating a lower fraction of inspired oxygen, reducing use of empiric inhaled nitric oxide (iNO), developing separate DR algorithms based on anticipated severity, and utilizing high frequency oscillatory ventilation as the initial mode of ventilation immediately after birth. With these temporal changes in management and introduction of an electronic medical record, infants were grouped into 3 epochs: 2008–2013, 2014–2018, and 2019–2023. Epochs were divided into roughly equal increments and marked by changes in clinical management as outlined below. Using the Clinical Outcomes Data Archive (CODA) Registry [16], we included all infants who received active treatment and excluded any infants with a planned palliative delivery. Outcome measures included extracorporeal membrane oxygenation (ECMO) therapy and survival. The CHOP Institutional Review Board approved this study (IRB 21-018553) with a waiver of informed parental consent.

### Hospital delivery room resuscitation protocols

#### Uniform interventions throughout all epochs: 2008–2023

**Fig. 1:**
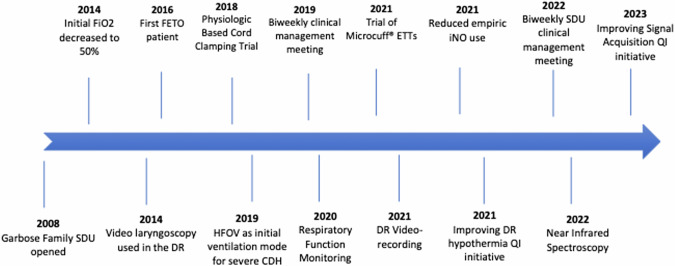
Timeline of changes in delivery room practice. DR delivery room, ETTs endotracheal tubes, FETO fetoscopic endoluminal tracheal occlusion, HFOV high frequency oscillatory ventilation, iNO inhaled nitric oxide, SDU Special Delivery Unit, QI quality improvement.

Throughout the entire study period, all infants with CDH were intubated immediately after birth, and received intermittent positive pressure ventilation (PPV) with a T-piece ventilator; settings included peak inspiratory pressures (PIPs) of 20–25 cm H_2_O, positive end expiratory pressure (PEEP) of 5 cm H_2_O, and a ventilation rate of 40–50 breaths/minute. A gastric decompression tube was placed immediately following intubation to promote adequate ventilation and stability by minimizing dilation of the intrathoracic bowel. Infants transitioned to a ventilator as soon as the endotracheal tube was secured. Vascular access was established and a blood gas, arterial if available, was used to guide ventilator adjustments (Fig. 1). Imaging and vascular access were obtained in the DR. It was our clinical practice to administer fentanyl with or without vecuronium to infants with significant hypoxia presumed to be secondary to pulmonary hypertension to improve pulmonary blood flow and reduce asynchrony with the ventilator. Throughout the study period, all new providers joining the SDU team participated in multidisciplinary delivery simulations focused on unique aspects of DR resuscitations of infants with surgical anomalies. The SDU DR team included a neonatologist, a neonatal fellow physician, an advanced practice provider, two to three neonatal intensive care unit (NICU) nurses, and a respiratory therapist. A pediatric surgeon frequently attended DR resuscitations.

Changes introduced to the resuscitation protocol throughout the 15-year study period are outlined with significant events highlighted in Fig. 1.

### Epoch 1: 2008–2013

#### Interventions

Following the opening of the SDU in 2008, infants with CDH were born within the freestanding children’s hospital, thereby eliminating the need for postnatal transfer after delivery. Initial resuscitation protocols were grounded in historical perspectives and expert consensus. In addition to the interventions above, protocols included intubation with a conventional laryngoscope and conventional mechanical ventilation with an initial FiO_2_ of 100%. Empiric iNO was used frequently prior to echocardiogram in the setting of profound hypoxia on 100% FiO2 and based on provider discretion.

#### Monitoring

Monitoring was limited to pulse oximetry and electrocardiography.

### Epoch 2: 2014–2018

#### Interventions

Beginning in 2014, initial DR FiO_2_ was decreased from 100% to 50%. FiO_2_ was then subsequently titrated to achieve pre-ductal oxygen saturation goals of ≥85% by 10 minutes of life and beyond. Riley et al. studied this practice change and demonstrated that an initial FiO_2_ of 50% was safe and effective [17]. Intubation in infants with CDH can be challenging due to significant airway deviation. Following review, our institutional experience demonstrated improved intubation outcomes with video laryngoscopy and in 2014 [18], video laryngoscopy was made standard of care.

In 2016, fetoscopic endoluminal tracheal occlusion (FETO) was introduced to our center for infants with severe left CDH. The Tracheal Occlusion to Accelerate Lung Growth trials found that FETO resulted in increased fetal lung volume and survival in infants with severe, isolated, left CDH with less clear benefit for infants with moderate CDH [19, 20]. In 2018, a pilot feasibility trial assessed physiologic based cord clamping, whereby the umbilical cord was clamped after intubation and lung aeration were established. Although physiologic based cord clamping was safe and feasible for infants with CDH, there was no clear benefit in clinical outcomes compared with historical controls [21]; therefore, this approach was not adopted for standard practice in our center.

### Epoch 3: 2019–2023

#### Interventions

Beginning in 2019, our DR protocol was updated to reflect two cohorts of infants with CDH based on anticipated severity, with severe CDH defined as an intrathoracic liver or right CDH. For infants with mild to moderate left CDH, initial conventional mechanical ventilation in the DR remained standard. Initial FiO2 was decreased further from 50% to 30% following close monitoring of infants started on 50% FiO2 with no adverse events noted and an overall reduction in exposure to supplemental oxygen. Patient level outcomes including delivery room outcomes (bradycardia, hypotension, Apgar scores) and NICU outcomes (survival, duration of invasive ventilation, incidence and duration of ECMO, days to surgery) were compared pre and post policy change to assess patient safety, as described in Riley et al. [17]. In contrast, as a response to high rates of hypercarbia in infants with severe CDH, high frequency oscillatory ventilation (HFOV) was established as the initial mode of ventilation for infants with severe CDH immediately after the endotracheal tube was secured; initial FiO2 remained at 50% for this population. Recommended HFOV settings include Mean Airway Pressure (MAP) of 11–13 mm H_2_O, amplitude of 30–35 adjusted to achieve appropriate chest wall vibration, and frequency or Hertz (Hz) of 6 for term infants that was increased with decreasing gestational age. Within these ranges, clinicians were encouraged to use the lowest possible settings to minimize barotrauma. The clinical impact of initial HFOV was assessed four years after this practice change; this analysis demonstrated significantly improved early gas exchange with no adverse differences in hospital outcomes among infants with severe CDH [22].

iNO was considered as an adjunctive DR therapy in infants predicted to have severe CDH with severe hypoxia on 100% FiO2. However, with a growing appreciation for left ventricle (LV) hypoplasia and dysfunction as a contraindication to iNO due to increased risk for pulmonary edema [23–25], we evaluated our experience and found increased ECMO need and higher mortality in infants with LV dysfunction that were treated with iNO [26]. Thus, in 2021 we changed our guidelines to recommend against use of iNO in infants with evidence of LV dysfunction.

We socialized these new guidelines and recommended obtaining an echocardiogram to evaluate LV function prior to starting iNO. Instead of empiric iNO use, we now use iNO on a case-by-case basis in a subset of infants with RV dysfunction without LV dysfunction and do not use iNO in the delivery room.

In 2021, we briefly introduced Microcuff® endotracheal tubes in the DR to allow inflation of the cuff peri-operatively to improve ventilation during the dynamic respiratory challenges during CDH repair. However, the practice was discontinued after an interim review of 22 patients revealed that more intubation attempts were needed to successfully intubate infants with CDH in the DR when Microcuff® tubes were used. Finally, in 2022, we undertook a quality improvement project to reduce rates of admission hypothermia for infants with congenital anomalies, given the evidence demonstrating both the association between admission hypothermia and increased morbidity and mortality in preterm infants and the increased risk of hypothermia in infants with congenital anomalies [27]. At baseline, 27% of infants were hypothermic (<36.5 degrees Celsius) on NICU admission. Plan, Do, Study Act cycles included standardizing the temperature of the DR and resuscitation bed, recommendations for increased frequency of temperature monitoring, trialing polyethylene lined hats, and implementing a DR thermoregulation checklist; in conjunction, these interventions led to an improvement with hypothermia seen in only 9% of infants in a recent cohort [28].

#### Monitoring

In 2020, we introduced enhanced monitoring in the DR beginning with respiratory function monitoring (RFM) to characterize the transitional pulmonary physiology of infants with CDH in real time during DR resuscitation [29]. RFM allows for evaluation of exhaled tidal volumes and end-tidal carbon dioxide monitoring during invasive positive pressure ventilation immediately after birth to guide ongoing ventilatory support. DR resuscitation video recording was also introduced in 2021 as routine practice for quality assurance [1]. In 2022, we introduced near infrared spectroscopy (NIRS) to evaluate cerebral hypoxia during the perinatal transition with ongoing evaluation of these data. In 2023, we started a quality improvement study to improve time to lead placement and vital sign acquisition in the DR given the unique challenges and competing interests of immediate intubation and invasive ventilation for infants with CDH. Plan, Do, Study, Act cycles have included transitioning to different electrocardiography leads with faster time to signal acquisition as well as rotating infants on the warmer bed to give bedside nurses improved access to placing leads on the infant.

#### Planning/team communication

In 2019, we implemented biweekly multidisciplinary CDH clinical management meetings to review DR and neonatal care. In 2022, we added alternating biweekly SDU management meetings to review upcoming deliveries and management plans, recent deliveries, and areas for education or improvement. In 2022, we implemented monthly video review conferences to identify facilitators and challenges to optimal stabilization that inform education initiatives, quality improvement projects, and guideline changes.

#### Main outcome measures

Main hospital-based outcome measures included extracorporeal membrane oxygenation (ECMO) therapy and survival.

### Data analysis

We evaluated the proportion of infants who experienced these outcomes across epochs. The Chi-squared test of trend was used to evaluate systematic increase or decrease in categorical variables across the time epochs. A linear regression model with the epochs coded as a continuous variable was used to evaluate trends in continuous variables. Additionally, a multivariable logistic regression was used to assess the confounder adjusted association between survival at NICU discharge and time. All analyses were conducted in R V.4.1.2.

## Results

**Fig. 2:**
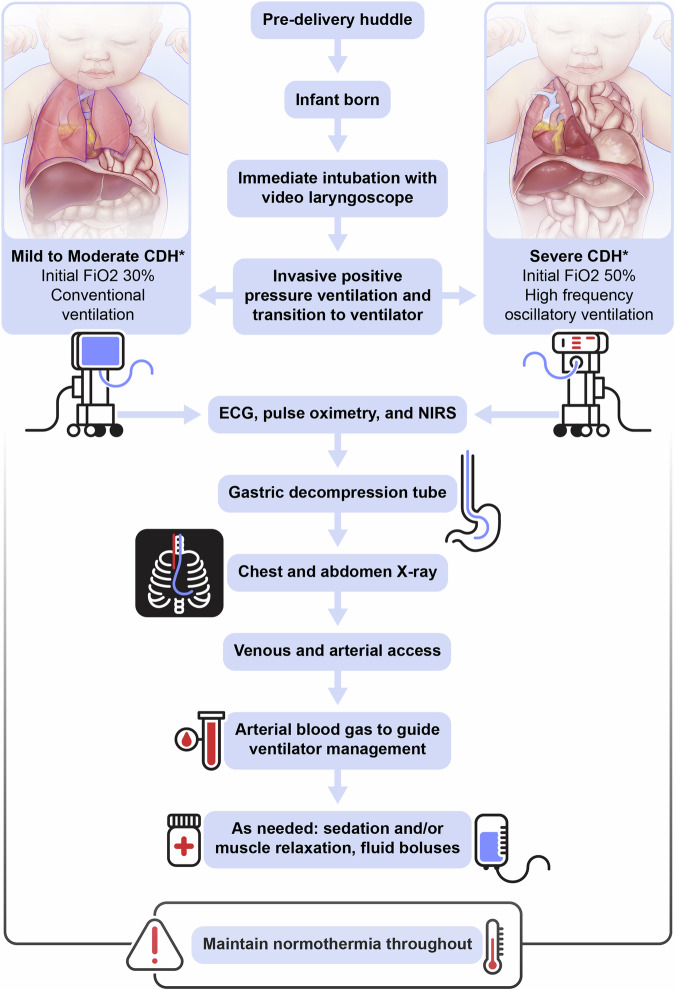
Severity-specific delivery room algorithm for the delivery room resuscitation of infants with CDH. ECG electrocardiogram, FiO2 Fraction of inspired oxygen, NIRS near infrared spectroscopy. *Mild to Moderate CDH = intrabdominal liver, Severe CDH = Intrathoracic liver or right CDH.

**Fig. 3:**
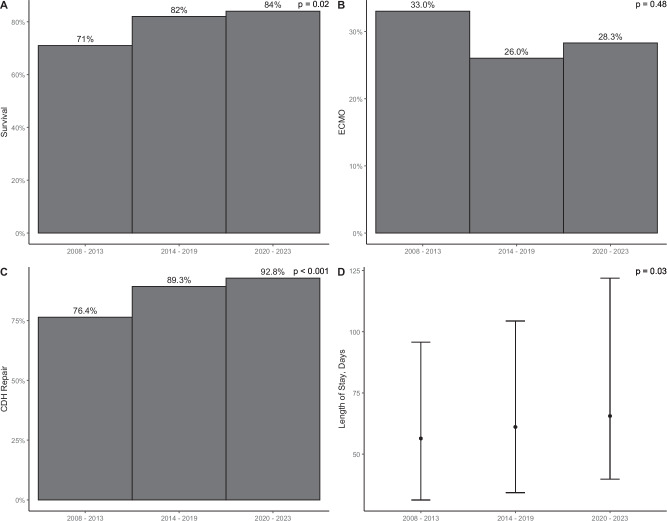
Survival, ECMO utilization, CDH repair, and length of stay compared across epochs. Survival (**A**), ECMO utilization (**B**), CDH repair (**C**) and length of stay (**D**). Survival improved significantly over time (**A**), from 71% (2008–2013) to 82% (2014–2018) and 83% (2019–2023), *p* = 0.02 for trend. There was no difference in ECMO utilization over time (**B**) but there was a significant increase in CDH repair (**C**), *p* < 0.001.

From 2008–2023, 454 infants with CDH were born in the SDU including 106 (2008–2013), 156 (2014–2018), and 192 (2019–2023) (Table 1). There was a trend toward increased severity across epochs. Differences in DR characteristics reflect temporal changes in DR management including lower initial fraction of inspired oxygen, two DR algorithms based on anticipated severity, and HFOV as the initial mode of ventilation after birth in infants with severe CDH (Table 2, Fig. 2). Tables 1 and 2 list the median and interquartile range for each variable, not the overall range. Despite increased severity, survival improved significantly over time, from 71% (2008–2013) to 82% (2014–2018) and 83% (2019–2023), *p* = 0.02 for trend. There was no difference in ECMO utilization over time, but there was a significant increase in CDH repair, *p* < 0.001 (Fig. 3). CDH repair details are shown in Supplementary Table 1.

**Table 1:** Cohort characteristics.

Characteristics	*n*(%), Mean ± SD, Median (IQR)			
	All 2008–2023 (*N* = 454)	Epoch 1 2008–2013 (*N* = 106)	Epoch 2 2014–2018 (*N* = 156)	Epoch 3 2019–2023 (*N* = 192)
Sex (Male)	264 (58.1%)	60 (56.6%)	91 (58.3%)	113 (58.9%)
CDH side (Left)	379 (83.5%)	90 (84.9%)	131 (84.0%)	158 (82.3%)
Liver position (Up)	298 (65.6%)	69 (65.1%)	101 (64.7%)	128 (66.7%)
LHR	*N* = 444 0.93 (0.72, 1.21)	*N* = 102 1.00 (0.80, 1.21)	*N* = 154 0.95 (0.75, 1.20)	*N* = 188 0.85 (0.66, 1.21)
O/E LHR^a,b^ (%)	*N* = 387 39.0 (29.0, 48.2)	*N* = 60 40.0 (34.0, 47.3)	*N* = 150 39.0 (29.0, 49.8)	*N* = 189 37.1 (28.1, 48.1)
Gestational age at time of imaging (weeks)	23.7 (22.0, 27.9)	23.5 (22.1, 28.4)	23.4 (22.0, 27.7)	24.2 (22.1, 27.7)
Mode of delivery (vaginal)	233 (51.3%)	54 (50.9%)	74 (47.4%)	105 (54.7%)
Gestational age at birth (weeks)	38.8 (37.6, 39.4)	38.8 (37.5, 39.4)	38.8 (37.8, 39.4)	38.7 (37.7, 39.4)
Birthweight (grams)	3040 (2740, 3370)	3010 (2760, 3270)	3070 (2810, 3430)	3040 (2690, 3370)
Off hours delivery (5 pm-8 am or weekends)	260 (57.3%)	56 (52.8%)	89 (57.1%)	115 (59.9%)
FETO	12 (2.6%)	0 (0%)	5 (3.2%)	7 (3.6%)

*FETO* fetoscopic endoluminal tracheal occlusion, *IQR* interquartile range, *LHR* lung to head ratio, *O/E* observed/expected, *SD* standard deviation.

^a^If N not presented, then available for all.

^b^For O/E LHR, trace method used when available, otherwise anterior/posterior (AP).

**Table 2:** Delivery room characteristics.

Characteristic	*n*(%), Median (IQR)			
	All 2008–2023 (*N* = 454)	Epoch 1 2008–2013 (*N* = 106)	Epoch 2 2014–2018 (*N* = 156)	Epoch 3 2019–2023 (*N* = 192)
1 min Apgar	5 (3, 8)	5 (3, 7)	5 (3, 7)	6 (4, 8)
5 min Apgar	8 (7, 9)	8 (6, 9)	8 (6, 9)	8 (7, 9)
Time to Pre-ductal SpO_2_ > 85% (minutes)	10.0 (6.0, 16.0)	9.0 (6.0, 16.0)	11.0 (7.0, 16.5)	9.0 (6.0, 15.0)
Time to HR > 100 beats per minute (minutes)	2.0 (1.0, 4.0)	1.0 (0, 2.0)	2.0 (1.0, 4.5)	3.0 (1.0, 4.5)
Time to intubation (minutes)	2.0 (1.0, 3.0)	2.0 (1.0, 3.0)	2.0 (2.0, 3.0)	2.0 (1.0, 3.0)
Ventilator type in delivery room CMV HFOV	*N* = 427 315 (73.8%) 112 (26.2%)	*N* = 96 94 (98.0%) 2 (2.0%)	*N* = 148 136 (92.0%) 12 (8.0%)	*N* = 183 85 (46.4%) 98 (53.6%)
First FiO_2_ in delivery room	50 (50, 80)	100 (100, 100)	50 (50, 50)	50 (50, 50)
Last FiO_2_ in delivery room	70 (50, 100)	100 (100, 100)	60 (50, 100)	50 (45, 100)
Maximum FiO_2_ in delivery room	100 (50,100)	100 (100, 100)	78 (50, 100)	70 (50, 100)
Fentanyl given in delivery room	301 (66.3%)	66 (62.3%)	102 (65.4%)	133 (69.3%)
Vecuronium given in delivery room	95 (20.9%)	8 (7.5%)	27 (17.3%)	60 (31.3%)
Normal saline bolus given in delivery room	173 (38.1%)	26 (24.5%)	67 (42.9%)	80 (41.7%)
Time in delivery room (minutes)	66.0 (57.0, 79.0)	64.0 (54.0, 73.0)	64.5 (53.0, 78.3)	70.0 (59.0, 80.5)
Time to first blood gas (minutes)	43.0 (35.0, 52.0)	42.5 (33.8, 48.3)	41.0 (33.5, 51.0)	45.5 (37.0, 54.0)
pH	7.11 (6.98, 7.21)	7.10 (6.96, 7.18)	7.06 (6.94, 7.15)	7.17 (7.05, 7.26)
CO_2_	72.6 (55.0, 92.6)	78.8 (60.5, 96.0)	81.5 (62.0, 105.0)	62.3 (49.0, 83.0)
PaO_2_	48.0 (40.0, 61.0)	47.5 (40.8, 60.3)	47.5 (41.0, 56.3)	48.0 (38.0, 65.3)
Base deficit	−8.0 (−11.0, −5.0)	−7.0 (−10.0, −6.0)	−9.5 (−12.0, −7.0)	−7.0 (−10.0, −4.0)
iNO in delivery room	5 (1%)	3 (2%)	2 (1%)	0 (0%)
iNO in first 24 h	217 (48%)	39 (37%)	85 (55%)	93 (48%)

*CMV* conventional mechanical ventilation, *HFOV* high frequency oscillatory ventilation, *iNO* inhaled nitric oxide, *IQR* interquartile range, *SD* standard deviation.

## Discussion

Management of infants with CDH continues to evolve. As large trials are lacking in this population, we reviewed the evolution of DR practice and outcomes in our large volume center. Notable changes to golden hour management for infants with CDH included lower initial fraction of inspired oxygen, severity-specific DR algorithms including HFOV as the initial mode of ventilation in severe CDH, and reduction of empiric inhaled nitric oxide. Despite increasing severity of illness among infants with CDH, survival to discharge improved over time with no difference in ECMO utilization. We believe this survival improvement is reflective of ongoing improvements we have implemented to ensure an experienced and collaborative team is available to respond to deliveries 24/7. While an ideal transition to postnatal life may help to optimize an infant’s overall course, it is the totality of CDH care (including the DR) that may explain improved survival outcomes.

There was a significant increase in CDH repair which likely reflects a more aggressive approach to infants with CDH in our center, despite increasing severity of illness. Over time, our approach to limitations of care changed to attempt stabilization, utilize ECMO if indicated, and attempt CDH repair prior to redirection of care if in line with a family’s goals of care. There has also been an increase in CDH repair of infants with concomitant complex congenital heart disease and other anomalies who would not have been ECMO candidates in the early years of this cohort. CDH repair is discussed on a case-by-case and was performed for infants with double outlet right ventricle, truncus arteriosus, and Tetralogy of Fallot in the later years of this cohort. Length of stay has increased over time, likely as a result of increased severity and an increase in CDH repair in the most severe infants.

Clinical trials, particularly randomized controlled trials, are rare in infants with CDH [19, 20, 30–34]. When attempted, these trials have been difficult to complete due to the rarity of CDH as well as high cost and low enrollment. Multicenter randomized controlled trials are even more challenging given the combination of a heterogeneous disease with a large spectrum of severity and significant practice variation across centers. Given these difficulties, CDH management is predominantly based on guidelines driven by expert opinion. As a result, large volume centers have a responsibility to evaluate and share observational data rigorously. While survival and CDH repair were higher than those reported by other groups, ECMO utilization was comparable [19, 20, 35]. As we have introduced changes to our golden hour management for this population, we have systematically studied these changes to ensure that expert-driven changes did not result in harm.

We have structured our CDH DR program using the framework of a Learning Health System [15] to inform and improve care. A Learning Health System begins with a learning community and is an iterative cycle to continuously improve care as we learn from each patient. We have multiple venues to review our data and revise our guidelines continuously including a team debrief after each delivery, weekly alternating CDH and SDU management meetings, and a monthly SDU video review meeting. Our interdisciplinary Clinical Outcomes Data Archive (CODA) Registry with data from both the electronic medical record and chart abstraction support this effort. To advance the care of infants with CDH without the benefit of clinical trial data, we employ a physiologic and data-driven approach to guide DR management. We have recently adopted advanced monitoring modalities such as NIRs and RFM to allow for real-time adaptation and individualization of DR resuscitations as needed. Finally, fetal interventions such as FETO have improved survival and decreased ECMO utilization for infants with severe CDH and have resulted in a different DR phenotype for infants on the most severe end of the CDH spectrum [36]. As FETO utilization and inclusion criteria increase, there may be an increased number of infants with this unique post-FETO phenotype that will need to be evaluated.

We acknowledge study limitations and unique strengths. This was an observational cohort study and therefore a non-randomized sample. We adjusted for known confounders, such as CDH side and severity in our analysis. As a single center study, outcomes may also reflect center-specific practice management differences. Therefore, we recommend that each center review their own key outcomes systematically as they make changes to CDH guidelines as our outcomes may not be generalizable.

Study strengths include one of the largest cohorts of infants with CDH born in a DR within a children’s hospital. DR practices and changes in management are not always represented in clinical trials; this study fills an important evidence gap specific to this high-risk population.

## Conclusion

Management of infants with CDH continues to evolve with ongoing experience and continual review of individual outcomes at our high-volume center. Despite increasing severity of illness, survival outcomes have improved over time. In the absence of clinical trial data, observational data should be evaluated within an established framework such as a Learning Health System to inform care in a data-driven manner.

## Supplementary information

Supplemental Table 1. CDH Repair Details

## Data Availability

The data that support the findings of this study are not publicly available due to privacy reasons but are available from the corresponding author upon reasonable request.
