# Supplementary material for: Golden hour management of infants with congenital diaphragmatic hernia: 15 year experience at a high-volume center
Source: J Perinatol. 2025 Feb 21;45(9):1247–54. doi: 10.1038/s41372-025-02226-z (PMC12431842; doi:10.1038/s41372-025-02226-z)
Supplement: Supplementary file 1 — Supplemental Table 1. CDH Repair Details [file 41372_2025_2226_MOESM1_ESM.docx]

| **Supplemental Table 1. CDH Repair Details** | | | | |
| --- | --- | --- | --- | --- |
| **Characteristic** | *n*(%), Median (IQR) | | | |
|  | All 2008-2023 (N=454) | Epoch 1 2008-2013 (N=106) | Epoch 2 2014-2018 (N=156) | Epoch 3 2019-2023 (N=192) |
| **Days to CDH repair** | 12 (6, 20) | 11 (5,18) | 15 (6,22) | 9 (5,17) |
| **ECMO status at time of CDH repair** No ECMO  Repair before ECMO  Repair on ECMO Repair after ECMO  Deceased before repair | 335 (74%)  1 (0%)  47 (10%)  53 (12%)  18 (4%) | 81 (76%)  0 (0%)  9 (9%)  11 (10%)  5 (5%) | 116 (74%) 0 (0%)  15 (10%) 20 (13%)  5 (3%) | 138 (72%) 1 (0%) 23 (12%) 22 (12%) 8 (4%) |
| ECMO=extracorporeal membrane oxygenation, IQR=interquartile range, SD=standard deviation. | | | | |
